# Rapidly expanding aortic root mycotic pseudoaneurysm with outflow tract fistula

**DOI:** 10.21542/gcsp.2021.15

**Published:** 2021-06-30

**Authors:** Ishan Parikh, Jeffrey Spindel, Mohammad Mathbout, Shahab Ghafghazi

**Affiliations:** 1 University of Louisville, Department of Internal Medicine, USA; 2 University of Louisville, Department of Cardiovascular Medicine, USA

## Abstract

We present a 50-year-old patient with chronic Stanford type-A aortic dissection, infective endocarditis, and rapidly expanding peri-aortic myocytic pseudoaneurysm with LVOT fistula. This case highlights the role of multimodality imaging in pathoanatomically complex-case evaluation.

## Case

**Figure 1.**
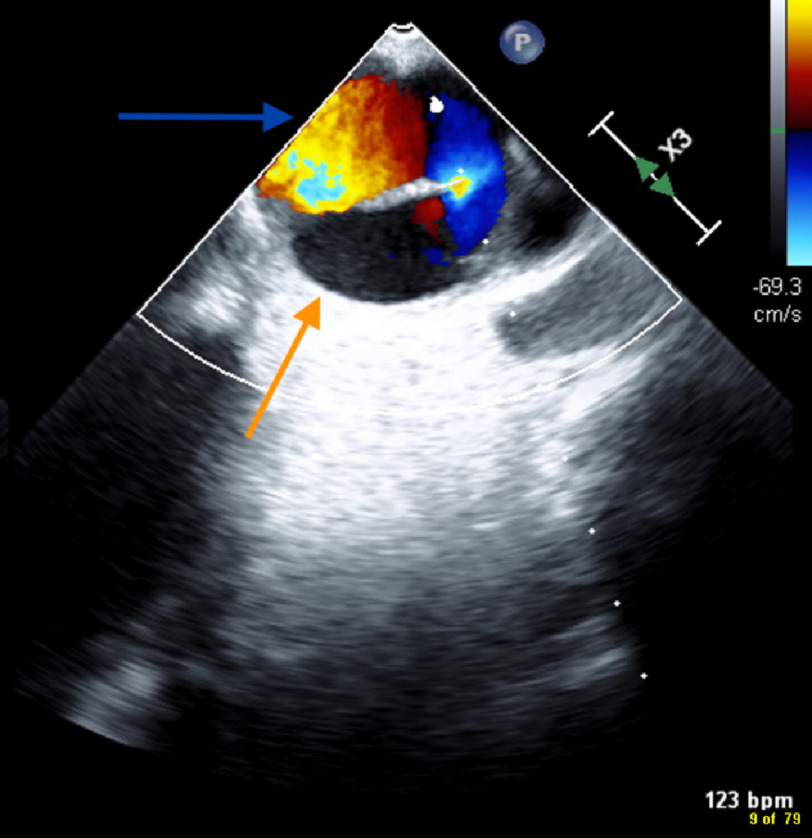
Chronic Stanford Type A aortic dissection on transesophageal echocardiogram with color flow doppler showing true lumen (blue arrow) and false lumen (orange arrow).

A 50-year-old male with a chronic Stanford type A aortic dissection (Figure 1), surgically repaired 10 years prior with an aortic conduit and a mechanical aortic valve, presented after a motor vehicle accident. Several injuries, intra-abdominal abscess, and persistent methicillin-resistant *Staphylococcus aureus* bacteremia with mechanical aortic valve endocarditis, prolonged his hospital stay.

**Figure 2.**
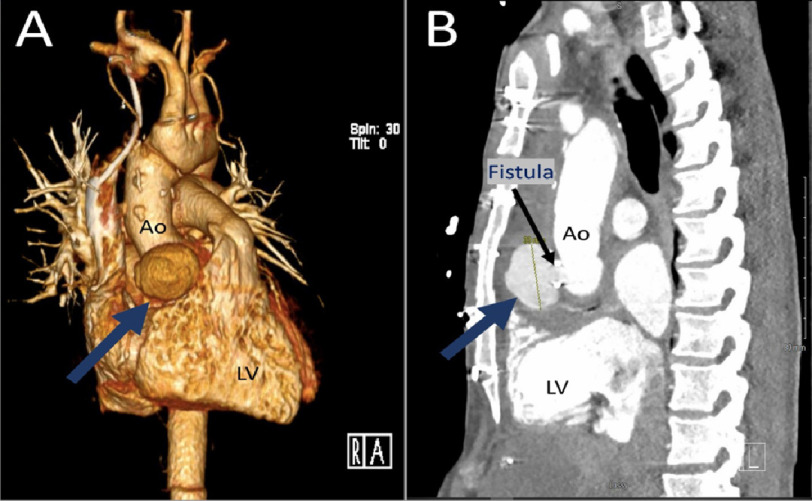
(A) 3-dimensional reconstruction of chest computerized tomography (CT) with blue arrow pointing to pseudoaneurysm. (B) Sagittal view of chest CT scan showing the same pseudoaneurysm with blue arrow, and fistulous connection as marked by black arrow.

Four weeks after the initial presentation, he had a non-ST elevation myocardial infarction (NSTEMI). Contrast enhanced chest computerized tomography (CT) revealed an irregular dilation measuring 6.2 × 3.2 × 3.6 cm distal to the reimplanted right coronary artery (RCA) (Figure 2), suggesting a pseudoaneurysm with a traumatic or infectious etiology.

**Figure 3.**
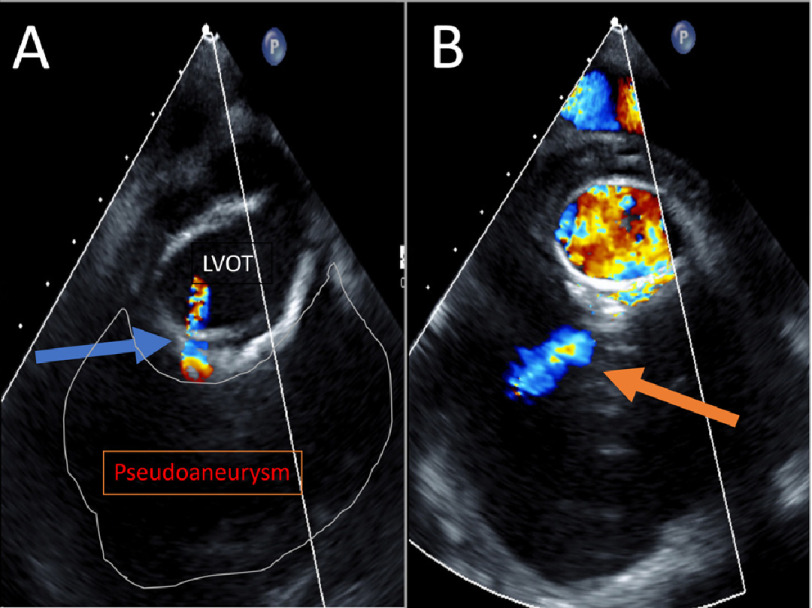
(A) TEE image with color flow doppler reveals flow between LVOT and pseudoaneurysm through a fistulous connection. (B) TEE image showing color flow doppler within pseudoaneurysm.

Transesophageal echocardiogram (TEE) demonstrated aortic valve endocarditis and an 8.9 × 7.7 × 5.2 cm fluid collection anterior to aortic root. Color Doppler indicated blood flow from left ventricular outflow tract (LVOT) to the cavity through a fistula (Figure 3).

**Figure 4.**
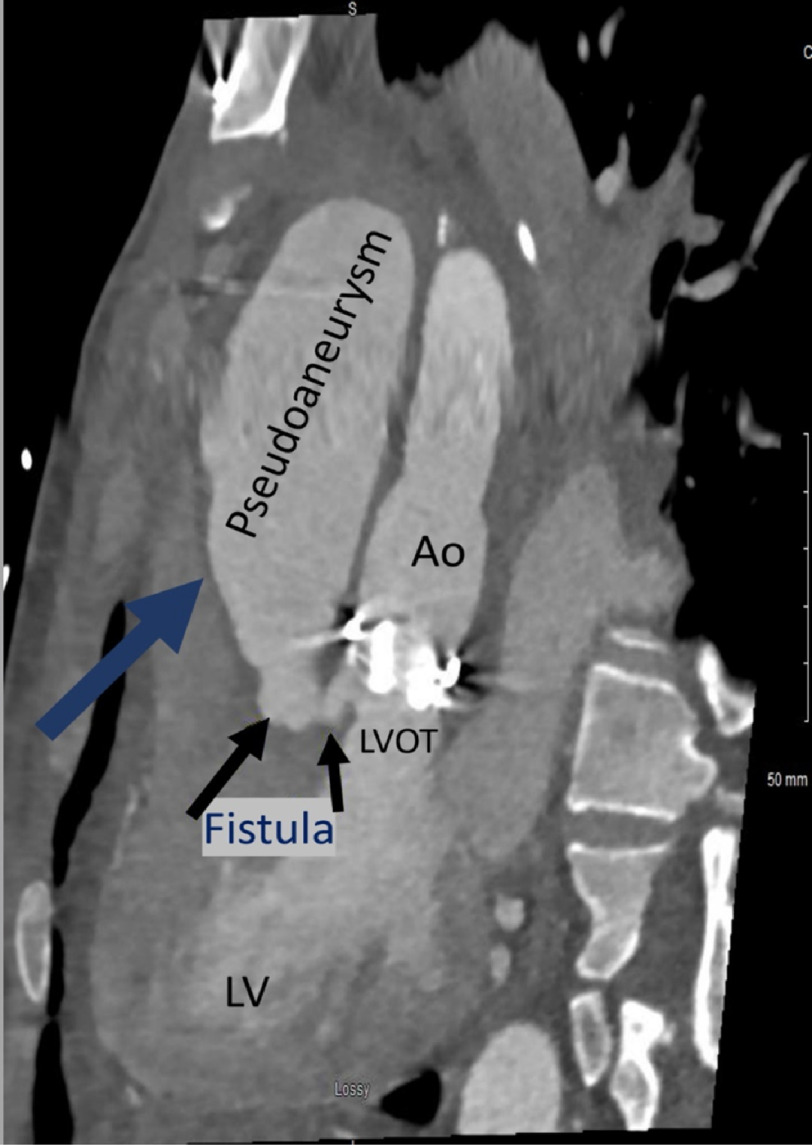
Dedicated coronary sagittal CT showing enlargement of the pseudoaneurysm with fistulous connection to LVOT.

Due to high operative risk, imaging was repeated a few days later, which revealed that the fluid collection had grown (Figure 4), and obstructed the proximal RCA, hence causing NSTEMI. Surgical exploration confirmed a large anterior mediastinal abscess (mycotic pseudoaneurysm), avulsion of the implanted RCA graft, and dehiscence of the mechanical valve from the aortic ring forming a fistula from LVOT to the abscess cavity. Open heart surgery was complicated by hemorrhage and disseminated intravascular coagulation, and the patient eventually passed away post-operatively.

## Discussion

Mycotic pseudoaneurysms are a known complication of bacterial endocarditis in prosthetic aortic valves, however, they are rarely reported in the literature^2^. Their tendency to expand rapidly and rupture make them highly life-threatening, and surgical intervention is often essential, despite high operative risk.

Our patient presented with a rare case of mycotic pseudoaneurysm due to persistent MRSA bacteremia, and mechanical valve endocarditis. Risk factors for pseudoaneurysm formation in this case included previous aortic valve and ascending aortic reconstruction, trauma, and endocarditis with persistent bacteremia^4,3^. If left untreated, such a pseudoaneurysm is at risk for rupture, tamponade, hemorrhagic shock, and sudden death^1^. Aortic pseudoaneurysms must be distinguished from true aneurysms as the former is at greater risk for rupture and immediate repair must be considered^1^.

Historically, aortic aneurysms and pseudoaneurysms were diagnosed by invasive angiography, but advances in non-invasive imaging have greatly improved diagnostic yield. Transthoracic echocardiography is frequently the initial test of choice and its utility has increased significantly with recent advances in 3D echocardiography which allows for better structural and vascular evaluations.

Similarly, TEE, enhanced by 3D imaging and Doppler, is well suited for assessment of aortic aneurysms and pseudoaneurysms, and allows for real-time assessment of blood flow, fistulae, and other findings^1^. In addition, magnetic resonance and CT imaging can further aid diagnosis and management.

In this case, TEE demonstrated to-and-fro blood flow between LVOT and the fluid collection (Figure 3), which is more typical of a pseudoaneurysm than a true aneurysm^1^. This correlated to the CT revealing a fistulous connection between LVOT and the pseudoaneurysm (Figures 2 and 4).

## Conclusion

This patient, with a past cardiac history, initially presented with trauma - however his NSTEMI required prompt evaluation and complex decision making. This case highlights the importance of using multimodality imaging to assess an expanding aortic fluid collection.
